# Education Research: Burnout and Perception of Value in a Cross-Section of Neurology Residency Program Directors

**DOI:** 10.1212/NE9.0000000000200144

**Published:** 2024-09-05

**Authors:** Alissa S. Higinbotham, James T. Patrie, Katherine B. Peters

**Affiliations:** From the Department of Neurology (A.S.H) and Department of Public Health (J.T.P.), University of Virginia School of Medicine, Charlottesville, VA; and Department of Neurology and Neurosurgery (K.B.P.), Duke University School of Medicine, Durham, NC.

## Abstract

**Background and Objectives:**

There are no dedicated studies specifically assessing burnout in neurology residency program directors (PDs). A study of residency and fellowship directors across specialties found neurology residency PDs had the highest work-related Copenhagen Burnout Inventory (CBI) score, which may reflect high-level burnout in neurology in general. Indeed, the American Academy of Neurology Burnout Task Force reported burnout in 60% of neurologists. The aims of this study are to determine the level of burnout in adult and pediatric neurology residency PDs, and to determine whether perception of value is related to burnout.

**Methods:**

In an IRB-approved study, 184 neurology residency PDs were emailed an anonymous survey consisting of the full CBI, demographic information, and 2 items addressing perception of value in the PD role: “I feel valued by my department in my educational role,” to which the participant could respond “strongly disagree, disagree, agree, or strongly agree,” and “satisfaction with current percent effort for the educational role,” to which the participant could respond “very dissatisfied, dissatisfied, satisfied, or very satisfied.” Data were analyzed through analysis of variance and ordinal logistic regression.

**Results:**

A total of 85 PDs (46%) responded to the survey. The average ± SEM personal, work-related, and patient-related CBI scores were 41.8 ± 2.2, 39.7 ± 2.2, and 32.4 ± 2.2, respectively. Higher personal and work-related CBI scores were associated with less positive perception of value (proportional odds ratio 1.03, 95% CI 1.01–1.06, *p* = 0.004 and proportional odds ratio 1.04; 95% CI 1.01–1.06, *p* = 0.003, respectively). Higher personal and work-related CBI scores were also associated with greater dissatisfaction with percent effort granted for the PD role. Level of burnout did not differ between adult and pediatric PDs. Burnout was not related to sex, years in practice, years as PD, academic rank, or percentage effort granted for the PD role.

**Discussion:**

Despite working in a field associated with high burnout, neurology residency PDs were found to have lower-level burnout than previously reported. Ninety-four percent of the PDs reported feeling valued in their role which was associated with lower-level personal and work-related burnout and may represent a key target for reflection and burnout intervention in the future.

## Introduction

The term, “burnout” first appeared in the medical field in the 1970s when Freudenberger, a psychologist, observed the phenomenon among health care volunteers in a free clinic and described it as a state of exhaustion related to “excessive demands on energy, strength, or resources” in the workplace.^1^ Over the years, it remains difficult to put the concept of burnout into words with a comprehensive definition. In 2021, experts from 29 countries reviewed 88 definitions of burnout. They agreed upon a consensus definition: “in a worker, occupational burnout or occupational physical and emotional exhaustion state is an exhaustion due to prolonged exposure to work-related problems.”^2^ While intentionally vague, this definition leaves much to be desired.

The challenges of defining burnout extend to assessing for it in individuals and populations. The most widely used method to assess burnout is the Maslach Burnout Inventory (MBI), a 22-item survey that measures burnout as a function of 3 domains: emotional exhaustion, depersonalization, and personal accomplishment. Each domain is scored separately using a frequency scale of never (0 points) to every day (6 points). Higher scores in emotional exhaustion and depersonalization and lower scores in personal accomplishment indicate higher concern for burnout. There were published cutoffs for what constitutes high burnout (emotional exhaustion ≥27, depersonalization ≥10, and personal accomplishment ≤33), but these were removed in more recent MBI versions to emphasize that burnout is on a continuum with its counterpart, engagement.^3,4^ Studies in the literature continue to use these and other cutoffs, however.

In 2018, Rotenstein et al. performed a systematic review of 182 studies on the prevalence of burnout in physicians; although 86% of the studies used the MBI, the prevalence of burnout varied from 0% to 80.5%. These authors concluded that varying survey methods, cutoffs, and definitions for burnout make it difficult to ascertain the prevalence of burnout in physicians. They recommend considering burnout as a continuous rather than dichotomous variable with surveys such as the Copenhagen Burnout Inventory (CBI) which are historically less susceptible to cutoffs and adaptations.^5^ The CBI is a widely used 19-item survey that measures burnout in 3 domains: personal, work-related, and client (patient)-related burnout. Items in each domain are scored as always (100), often (75), sometimes (50), seldom (25), or never/almost never (0). The mean for each domain is calculated for a score from 0 to 100. Higher scores indicate higher levels of burnout.^6^

Despite the challenges of defining burnout and its prevalence, there is increasing discussion in the literature on the implications of burnout in medical education including poor patient outcomes, physician and trainee mental health concerns, and job turnover.^1,7^ However, a critical gap remains: burnout among residency program directors (PDs). Residency PDs are some of the most influential mentors in trainees' careers, but symptoms of burnout in PDs could “trickle down” to impressionable trainees. A study of 756 residents found higher burnout in those who did not rate their residency program leadership favorably on items such as “holds career development conversations with me” and “encourages me to develop my talents and skills.”^8^

In the limited available literature on burnout in PDs, some studies report similar rates of burnout, while others report relatively higher rates. A study of 282 internal medicine PDs used a 2-item MBI and found high emotional exhaustion and depersonalization (defined as symptoms at least weekly) in 27% and 10.4% of PDs, respectively, and overall burnout (not defined) in 28.7% compared with non-PD physicians at 37.9%, 29.4%, and 45.5%, respectively.^9^ A study of 245 family medicine PDs also used a 2-item MBI and found high emotional exhaustion and depersonalization (defined as symptoms at least weekly) in 27.3% and 15.8% of those PDs, respectively.^10^ A study of 191 general surgery PDs used the full MBI found high emotional exhaustion in 25% and low personal accomplishment in 17%, comparable with national norming data of health care providers. High depersonalization rates were lower in these PDs at 13% compared with normative data.^11^

Conversely, a study of 100 anesthesiology residency PDs used a 12-item MBI and found burnout (defined as emotional exhaustion ≥26, depersonalization ≥10, and personal accomplishment <32) in 21% with an additional 31% at high risk for burnout based on moderate to high MBI scores. These PDs were also more likely to report lower job satisfaction and desire to resign.^12^ A study of 210 psychiatry residency PDs used a 1-item MBI and found burnout (defined as symptoms almost daily or once a week) in 44%, 77% of whom expressed a desire to resign.^13^

Dedicated studies of burnout in neurology resdiency PDs are under-represented in the literature. However, 1 study of 904 residency and fellowship directors across specialties, including 37 neurology residency PDs, found neurology residency directors had the highest work-related CBI score (63.0 on the 100-point scale); the third highest personal CBI score (61.5), after neurology fellowship directors and obstetrics and gynecology residency directors; and the third highest patient-related CBI score (37.8).^14^ Although the sample size was small and more studies are needed, this is an alarmingly high level of burnout that may reflect the alarmingly high level of burnout in the specialty of neurology in general rather than in the role of PD. Indeed, in 2016, the American Academy of Neurology Burnout Task Force surveyed more than 4,000 neurologists with the MBI and found burnout (defined as emotional exhaustion ≥27 or depersonalization ≥10) in 60% of participants.^15^

The aims of this study are to determine the level of burnout in neurology residency PDs and whether perception of value in the PD role is associated with the burnout level. Perception of value is not examined explicitly in PD burnout studies, but factors previously found to be associated with PD burnout, such as full-time equivalent support, chair support, and autonomy,^11,12^ share a similar overarching theme of perception of value in the role. Furthermore, a concept similar to perception of value—sense of mattering, a psychosocial construct comprising awareness, reliance, importance, and ego extension—is negatively associated with burnout in nurses.^16^

## Methods

### Study Questionnaire

A 27-item anonymous survey was designed (eAppendix 1). Six items addressed demographic information including sex, years in practice, academic rank, educational role (adult or pediatric neurology residency PD), years in role, and percent effort allocated to the role. Two items addressed perception of value in the PD role: “I feel valued by my department in my educational role,” to which the participant could respond “strongly disagree, disagree, agree, or strongly agree,” and “satisfaction with current percent effort for the educational role,” to which the participant could respond “very dissatisfied, dissatisfied, satisfied, or very satisfied.” The remaining 19 items assessed burnout with the full CBI. The personal, work-related, and client (patient)-related items were mixed to avoid stereotyped responses in each category.

The authors used the National Resident Matching Program data from 2019 to 2023 to gather a list of active adult and pediatric neurology residency programs. As of 2023, there were 180 adult and 80 pediatric neurology residency programs. The authors then conducted a manual Internet search for the PDs' email addresses and/or Twitter accounts and found contact information for 190 of the 260 PDs (184 email addresses and 6 additional active Twitter accounts). Participants were eligible for the survey if they were an adult or pediatric neurology residency PD in the academic year of 2022–2023. Assistant/associate PDs were not eligible for the survey given the variability of their roles across institutions. The survey was entered into Duke Qualtrics and distributed through an email link to 184 adult and pediatric neurology residency PDs on July 7, 2023. The recipients could forward the email to the appropriate party if they were not PD during the 2022–2023 academic year. One reminder email was sent 2 weeks later. A link to the survey was also posted on social media through the Twitter platform.

### Standard Protocol Approvals

This study was approved by the Duke Institutional Review Board.

### Data Analyses

Survey participant characteristics and opinions were summarized by frequencies and relative frequencies. Participant personal, work-related, and patient-related CBI scores were summarized by the mean, SD, median, interquartile range, and range of the CBI score empirical distribution.

As primary analyses, 2 sets of analyses of variances (ANOVAs) were conducted. One set of ANOVAs focused on determining whether significant variability in personal CBI scores, work-related CBI scores, and patient-related CBI scores is explained by the response (strongly agree, agree, or disagree) to the statement “I feel valued by my department in my educational role.” The second set of ANOVAs focused on determining whether significant variability in personal CBI scores, work-related CBI scores, and patient-related CBI scores is explained by the response (very dissatisfied or dissatisfied, satisfied, very satisfied) to level of “Satisfaction with current percent effort for the education role.”

As secondary analyses, ANOVA was used to determine whether significant variability in personal CBI scores, work-related CBI scores, and patient-related CBI scores is explained by sex, academic rank, educational role (adult or pediatric neurology residency PD), length of time in the educational role, and percent effort allocated to the role.

For the primary and secondary ANOVAs, the hypothesis testing strategy was the same. First, a global *F* test was conducted to determine whether the means of the CBI score distributions are equal for the different levels of the ANOVA independent variable. If this test was rejected at the α = 0.05 significance level, then the means of the CBI score distributions were compared through a Welch *t* test in a pairwise manner to determine which CBI mean scores differ at the α = 0.05 level after Bonferroni type I error rate correction. The MIXED procedure of SAS version 9.4 (SAS Institute Inc., Cary, NC) was used to conduct the ANOVAs.

As additional secondary analyses, the relationship between the participants' responses (strongly agree, agree, or disagree) to the statement “I feel valued by my department in my educational role” and the participants' personal CBI scores, work-related CBI scores, and patient-related CBI scores were examined by ordinal logistic regression.

### Data Availability

Anonymous data not published in this article can be made available by request from any qualified investigator.

## Results

**Figure 1:**
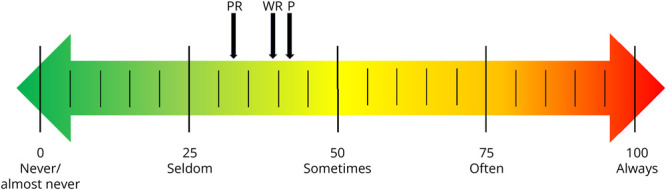
Mean Burnout Scores on the CBI Continuum Down facing arrows indicate the mean personal (P), 41.8 (95% CI 37.5–46.1); mean work-related (WR), 39.7 (95% CI 35.4–43.9); and mean patient-related (PR), 32.3 (95% CI 27.9–36.6) Copenhagen Burnout Inventory (CBI) scores for 85 neurology residency program directors placed in the context of a burnout continuum. Corresponding median CBI scores are 41.7 (IQR 29.2–58.3), 39.3 (IQR 28.6–53.6), and 29.2 (IQR 16.7–45.8), respectively. CBI = Copenhagen Burnout Inventory; IQR = interquartile range.

Ninety-nine PDs (54%) started the survey, but 14 were deemed nonresponders based on incomplete demographic information and failure to complete the CBI. Eighty-five PDs (46%) were responders and included in the data analysis. The personal and professional characteristics of the 85 participants are summarized in Table 1. Forty-two percent were male, 50% were 10 years or less out from training, 56% were adult neurology residency PDs, and 56% had been PD for 5 years or less. The majority (87%) were satisfied with their percentage effort granted for the PD role, and the majority (94%) reported feeling valued by their department in their PD role. Personal, work-related, and patient-related CBI scores are summarized in Table 2, and Figure 1 identifies where the mean CBI scores fall along the CBI score spectrum. Overall, 36 of 85 PDs (42%) had an average CBI score >50 (symptoms more than sometimes), but only 12 of 85 PDs (14%) had an average CBI score ≥75 (symptoms often to always) in 1 or more categories of burnout (personal, work-related, or patient-related).

**Table 1:** Participant Characteristics

Characteristics	No. of responders (%) n = 85
Sex	
Male	36 (42)
Female	49 (58)
Other	0 (0)
No response	0 (0)
Years in practice (not including training)	
0–5	12 (14)
6–10	31 (36)
11–20	24 (28)
21–25	10 (12)
26–30	4 (5)
31–40	3 (4)
>40	0 (0)
No response	1 (1)
Academic rank	
Assistant professor	25 (29)
Associate professor	35 (41)
Professor	22 (26)
No response	3 (4)
Educational role	
Adult neurology residency program director	48 (56)
Pediatric neurology residency program director	36 (42)
No response	1 (1)
Years as program director	
0–5	48 (56)
6–10	22 (26)
11–20	12 (14)
21–25	2 (2)
No response	1 (1)
I feel valued by my department in my educational role	
Strongly disagree	0 (0)
Disagree	5 (6)
Agree	32 (38)
Strongly agree	48 (56)
No response	0 (0)
Percent effort granted for educational role	
16%–20%	10 (12)
21%–25%	5 (6)
26%–30%	10 (12)
31%–35%	9 (11)
36%–40%	15 (18)
41%–45%	4 (5)
46%–50%	21 (25)
>50%	10 (12)
No response	1 (1)
Satisfaction with current percent effort for educational role	
Very dissatisfied	3 (4)
Dissatisfied	8 (9)
Satisfied	47 (55)
Very satisfied	27 (32)
No response	0 (0)

Frequencies and relative frequencies of program director characteristics with respect to sex, years in practice, academic rank, educational role, years as a program director, perception of value by department in the educational role, percent effort granted for the educational role, and satisfaction with current percent effort for educational role.

**Table 2:** Burnout Scores

CBI score	n	Mean	SD	Median	IQR	Range
Personal	85	41.8	20.1	41.7	29.2, 58.3	0, 83.3
Work-related	85	39.7	19.8	39.3	28.6, 53.6	0, 89.3
Patient-related	85	32.3	20.0	29.2	16.7, 45.8	0, 79.2

Abbreviations: CBI = Copenhagen Burnout Inventory; IQR = the interquartile range of the distribution (25th, 75th percentiles); mean = the mean of the distribution; median = median of the distribution; N = sample size; range = the range of the distribution (minimum, maximum).

CBI personal, work-related, and patient-related score empirical distribution summaries.

### I Feel Valued by My Department in My Educational Role

**Figure 2:**
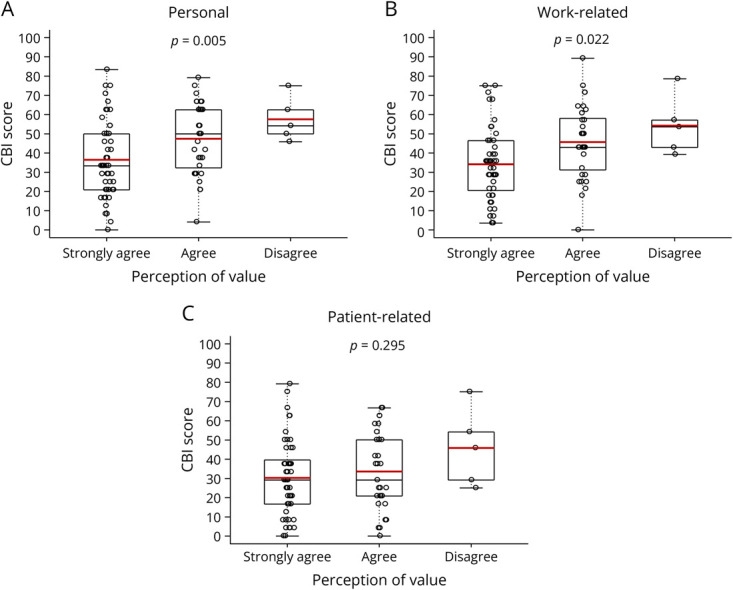
Relationship Between Burnout and Perception of Value Empirical distributions for personal CBI score (A), work-related CBI score (B), and patient-related CBI score when stratified by perception of value in the education role that is, “I feel valued by my department in my educational role. Red horizontal line within the interior of the box in the box and whisker plot identifies the mean of the empirical distribution. Black horizontal line within the interior of the box in the box and whisker plot identifies the median of the empirical distribution. *p* Values are for the global ANOVA test that all CBI mean scores are equal. ANOVA = analysis of variance; CBI = Copenhagen Burnout Inventory.

The relationships between the participants' personal, work-related, and patient-related CBI scores and responses (strongly agree, agree, disagree) to the statement “I feel valued by my department in my educational role” are displayed in Figure 2, A–C, respectively.

Personal CBI mean scores differed depending on how the participant responded (strongly agree, agree, and disagree) to the statement “I feel valued by my department in my educational role” (*p* = 0.005). The mean personal CBI score was 36.5 (95% CI 30.6–42.3) for the participants who “strongly agree,” 47.4 (95% CI 40.7–54.0) for the participants who “agree,” and 57.5 (95% CI 43.1–71.9) for the participants who “disagree” with the statement. Participants who “strongly agree” had a lower mean CBI score than the participants who “agree” (*p* = 0.044) and “disagree” (*p* = 0.029) with the statement. Personal CBI mean scores did not differ between the participants who “agree” and “disagree” with the statement (*p* = 0.416).

Work-related CBI mean scores also differed depending on how the participants responded (strongly agree, agree, and disagree) to the statement “I feel valued by my department in my educational role” (*p* = 0.022). The mean work-related CBI score was 34.2 (95% CI 28.6–39.7) for the participants who “strongly agree,” 47.7 (95% CI 38.8–52.6) for the participants who “agree,” and 54.3 (95% CI 35.1–73.5) for the participants who “disagree” with the statement. Participants who “strongly agree” had a lower mean CBI score than the participants who “agree” (*p* = 0.029). Participants who “strongly agree” also had a lower mean CBI score than those who “disagree” with the statement before Bonferroni type I error rate correction (*p* = 0.039), but not after (*p* = 0.118). Work-related CBI mean scores did not differ between participants who “agree” and “disagree” with the statement (*p* = 0.919).

Patient-related CBI mean scores did not differ between respondents who “strongly agree,” “agree,” and “disagree” with the statement “I feel valued by my department in my educational role” (*p* = 0.295). The mean patient-related CBI score was 30.3 (95% CI 24.5–36.0) for the participants who “strongly agree,” 33.6 (95% CI 26.2–41.0) for those who “agree,” and 45.8 (95% CI 20.8–70.9) for participants who “disagree” with the statement.

### Satisfaction With Current Percent Effort for Education Role

**Figure 3:**
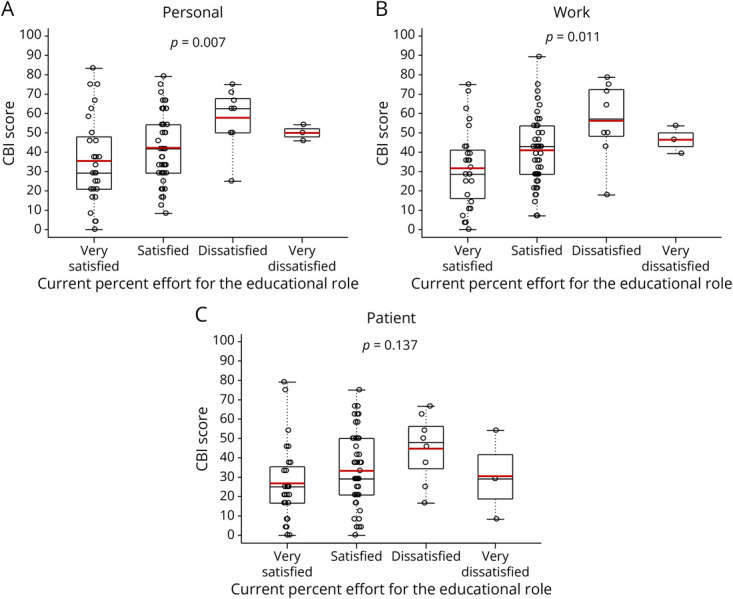
Relationship Between Burnout and Satisfaction With Effort for the PD Role Empirical distributions for personal CBI score (A), work-related CBI score (B), and patient-related CBI score when stratified by the participants opinions about “Satisfaction with current percent effort for educational role.” Red horizontal line within the interior of the box in the box and whisker plot identifies the mean of the empirical distribution. Black horizontal line within the interior of the box in the box and whisker plot identifies the median of the empirical distribution. *p* Values are for the global ANOVA test that all CBI mean scores are equal. ANOVA = analysis of variance; CBI = Copenhagen Burnout Inventory; PD = program director.

The relationships between the participants' personal, work-related, and patient-related CBI scores and responses (very dissatisfied, dissatisfied, satisfied, or very satisfied) to the level of “Satisfaction with current percent effort for education role” are displayed in Figure 3, A–C, respectively. A limited number of participants (n = 3) responded “very dissatisfied,” so “very dissatisfied” and “dissatisfied” were merged into 1 category, “very dissatisfied or dissatisfied,” for the ANOVA.

Personal CBI mean scores differed depending on how the participant responded (very dissatisfied or dissatisfied, satisfied, and very satisfied) to the level of “Satisfaction with current percent effort for education role” (*p* = 0.007). The mean personal CBI was 55.7 (95% CI 46.3–65.1) for those were “very dissatisfied or dissatisfied,” 42.2 (95% CI 36.9–47.5) for those who were “satisfied,” and 35.5 (95% CI 26.4–44.5) for those who were “very satisfied” with their current percent effort for the PD role. Participants who were “very dissatisfied or dissatisfied” had a higher personal CBI mean score than participants who were “satisfied” (*p* = 0.042) and “very satisfied” (*p* = 0.007). Personal CBI mean scores did not differ between participants who were “satisfied” and “very satisfied” (*p* = 0.598) with their current percent effort for the PD role.

Work-related CBI mean scores also differed depending on how the participant responded (very dissatisfied or dissatisfied, satisfied, and very satisfied) to the level of “Satisfaction with current percent effort for education role” (*p* = 0.011). The mean work-related CBI score was 53.6 (95% CI 41.6–65.6) for those who were “very dissatisfied or dissatisfied,” 41.0 (95% CI 35.7–46.3) for those who were “satisfied,” and 31.7 (95% CI 23.7–39.7) for those who were “very satisfied” with their current percent effort for the PD role. Participants who were “very dissatisfied or dissatisfied” had a higher work-related CBI mean score than participants who were “very satisfied” (*p* = 0.010). Participants who were “very dissatisfied or dissatisfied” had a work-related CBI mean score that was borderline statistically higher than those who were “satisfied” before Bonferroni type I error rate correction (*p* = 0.054), but not after (*p* = 0.161). Similarly, participants who were “satisfied” had a work-related CBI mean score that was borderline statistically higher than respondents who were “very satisfied” before Bonferroni type I error rate correction (*p* = 0.052), but not after (*p* = 0.157).

Patient-related CBI mean scores did not differ depending on how the participants responded (very dissatisfied or dissatisfied, satisfied, and very satisfied) to their level of “Satisfaction with current percent effort for education role” (*p* = 0.137). The mean patient-related CBI score was 40.9 (95% CI 28.1–53.7) for those who were “very dissatisfied or dissatisfied,” 33.03 (95% CI 27.5–39.1) for those who were “satisfied,” and 26.9 (95% CI 18.9–34.8) for those who were “very satisfied.”

### Supplemental Results Materials

A complete set of summary statistics for the personal, work-related, and patient-related CBI scores stratified by response to the statements “I feel valued by my department in my educational role” and “Satisfaction with current percent effort for educational role” is provided in Supplemental Materials eTable 1a and eTable 2a, respectively. Corresponding ANOVA summaries are provided in eTables 1b-d and eTables 2b-d, respectively.

### Sex, Academic Title, Educational Role, Years as PD and Percent Effort Granted for PD Role

eFigures 1–5 show the empirical distributions of the personal, work-related, and patient-related CBI scores with the CBI score distributions stratified by sex (female, male), academic title (assistant professor, associate professor, professor), educational role (adult or pediatric neurology residency PD), years as PD (0–5, 6–10, >10), and percent effort granted for educational role (<20%, 21%–30%, 31%–40%, 41%–50%, and >50%), respectively. Corresponding summary statistics are provided in eTables 3a, 4a, 5a, 6a, 7a, respectively. ANOVA summaries are presented in eTables 3b-d, 4b-d, 5b-d, 6b-d, and 7b-d, respectively. Sex, academic title, educational role, years as PD, and percent effort for educational role were not a significant source of personal CBI score variability (*p* = 0.266, 0.409, 0.807, 0.935, and 0.170, respectively), work-related CBI score variability (*p* = 0.650, 0.499, 0.696, 0.898, and 0.470, respectively), or patient-related CBI score variability (*p* = 0.234, 0.615, 0.371, 0.832, and 0.748, respectively).

## Discussion

This study addressed the largely unanswered question of burnout among neurology residency PDs. The mean personal, work-related, and patient-related CBI scores were lower in this study than previously reported in 1 small sample of 37 neurology residency PDs (41.8 vs 61.5, 39.7 vs 63.0, and 32.4 vs 37.8, respectively).^14^ This observation could be partially attributed to the finding that most PDs in this study (94%) reported feeling valued by their department in their role, which was associated with significantly lower-level personal and work-related burnout.

Previously reported PD-specific factors associated with higher burnout risk include Accreditation Counsel for Graduate Medication Education and compliance issues,^12^ balancing administrative roles,^11,13^ perceived lack of effectiveness in the PD role,^11,12^ discrimination in the role,^13^ perceived lack of control over events and schedules,^11^ lack of adequate time for the role,^11^ disputes with the chair,^12^ and fewer years in the PD role.^11^ In contrast to the latter, however, 3 studies of PD burnout did not find an association between PD tenure and burnout,^7,9,12^ and our study did not find an association between years in the PD role and burnout. PD-specific factors previously reported to be unrelated to burnout include program size^9,11,12^ and trainee board pass rate.^7,9^

Personal factors previously reported in the literature for PDs associated with higher risk of burnout include less spousal/significant other support,^9,12^ younger age,^9,11^ lack of work-life balance,^10^ and financial issues.^10^ Less resilience^17^ and health issues^18^ are personal factors associated with burnout in physicians in general. Number of children at home,^10^ vacation days taken,^10^ and sex^7,9,12^ were previously not associated with burnout in PDs. Similarly, we did not find an association between sex and PD burnout.

Previously reported physician-related factors associated with greater burnout include more hours worked per week,^9,15^ more outpatients seen per week,^15^ more nights on call per week,^15^ greater burden of clerical work,^13,15^ ineffective leadership,^18^ negative work culture,^18^ and schedule inflexibility.^18^ Effective support staff, job autonomy, and meaningful work are associated with lower burnout.^15^ Regarding the latter factor, meaningful work, spending less than 20% effort on the activity, one finds most meaningful in academic practice is associated with higher burnout.^19^ In this study, assuming PDs find their role meaningful, percentage effort granted for the PD role, even when above 20% was not associated with burnout. It is difficult to interpret the effect of percentage effort for the PD role specifically without accounting for program size, number and level of involvement of assistant/associate PDs, involvement of chief residents, and numerous other factors. Thus, the more relevant question is not the percentage of effort, but rather satisfaction with that percentage effort, which was significantly associated with burnout in this study. This finding was similar to a previous study in which inadequate time for the PD role was associated with higher burnout.^11^

**Figure 4:**
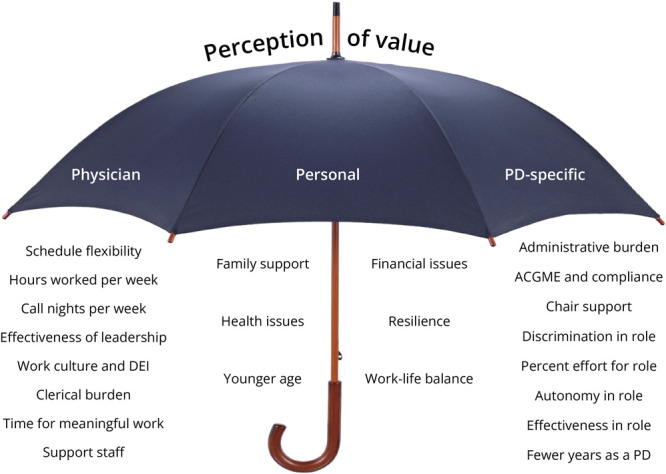
Perception of Value as an Umbrella for Burnout Factors Many of the previously reported physician-related, personal, and PD-specific burnout-contributing factors could fall under the broader theme of perception of value. Rather than attempting to navigate countless burnout factors, this figure provides a more manageable approach to burnout reflection. ACGME = Accreditation Counsel for Graduate Medication Education; DEI = diversity, equity, and inclusion; PD = program director.

It is impractical and frankly impossible to capture all the aforementioned plus the yet-to-be-determined factors that contribute to burnout in PDs as a group, let alone in an individual PD's unique situation, through research surveys. Thus, this study approached burnout more broadly by assessing perception of value, which in the authors' opinion is an underexplored “umbrella factor” for many burnout-contributing factors and concepts (Figure 4). Rather than attempting to navigate countless burnout factors, Figure 4 offers PDs a more manageable approach to burnout. For example, if a PD does not feel valued by his department in his role and after reflection on why that is, determines he does not have adequate time to execute his vision for the residency program, then armed with the findings from the current and previous studies,^11,15,19^ he could address effort renegotiation with his chair.

This study has several limitations. First, this study was a cross-sectional evaluation of burnout in PDs at only 1 point in time (July of the academic year). The level of burnout could fluctuate as the year progresses. Ideally, CBI scores would be assessed over time to evaluate stability. Second, we were unable to find contact information for 70/260 PDs, and although they may have encountered the survey link on social media, this limited our sample size and potentially generalizability of results. Of the PDs we were able to sample, our survey response rate of 46% is in line with the average for online educational research surveys at 44%.^20^ Third, we do not have demographic information on the 85 PDs who chose not to respond to the survey invitation, and the demographic information for the 14 nonresponders who partially completed the survey was too incomplete for meaningful analysis. A responder bias may be present in which those who responded were less burnt out and thus more willing to take the time to participate in the survey. One could imagine the alternative, however, in which those experiencing burnout would be more likely to participate to bring the issue of burnout in PDs to light.

Lastly, is a limitation of burnout research in general. Most studies report prevalence of “high burnout” as a percentage by using various burnout score cutoffs.^5^ In this study, we avoided arbitrary cutoffs as is now recommended by the MBI and CBI.^3-6^ Instead, we reported the average burnout scores on the continuum in Figure 1 to demonstrate that, on average, neurology residency PDs experience personal, work-related, and patient-related burnout seldomly to sometimes.

To determine the prevalence of PDs who experienced “high burnout” as in other studies, we would need to define a cutoff. For example, if we chose to define “high burnout” as symptoms more than sometimes in at least 1 category of burnout (personal, work-related, or patient-related), 42% of the neurology residency PDs in this study would have “high burnout.” This is lower than the 60% of neurologists determined to have burnout (high emotional exhaustion or high depersonalization on the MBI) by the AAN Burnout Task Force,^15^ but these findings are not directly comparable because different surveys were used. If we defined “high burnout” as experiencing symptoms between often and always in at least 1 burnout category, then only 14% of neurology residency PDs would have “high burnout.” By changing the cutoff definition of “high burnout,” our findings would vary from 14% to 42%; thus, we did not select a cutoff.

This study achieved its first aim to define the level of burnout among neurology residency PDs. The next step for future studies is to determine whether the burnout level is stable over time by surveying PDs at the midpoint of the academic year and longitudinally over the years. This study also achieved its aim to demonstrate a relationship between burnout level and perception of value. The next step will be to determine what interventions will be most effective to increase perception of value in the role of PD. A focus group and thematic analysis of PDs using Figure 4 to guide reflection on factors and interventions that have been successful in the past or could be successful in the future to increase value could be quite informative in that regard. The results of the present and future studies could then be shared with leadership to drive departmental and institutional change that recognizes the incredible value of neurology residency PDs.

## Appendix Authors

**Table:**

Name	Location	Contribution
Alissa S. Higinbotham, MD	Department of Public Health, University of Virginia School of Medicine, Charlottesville	Drafting/revision of the manuscript for content, including medical writing for content; major role in the acquisition of data; study concept or design; analysis or interpretation of data
James T. Patrie, MS	Department of Public Health, University of Virginia School of Medicine, Charlottesville	Drafting/revision of the manuscript for content, including medical writing for content; analysis or interpretation of data
Katherine B. Peters, MD, PhD	Department of Neurology and Neurosurgery, Duke University School of Medicine, Durham, NC	Drafting/revision of the manuscript for content, including medical writing for content; major role in the acquisition of data; study concept or design; analysis or interpretation of data

## Glossary

ANOVA: analysis of variance
CBI: Copenhagen Burnout Inventory
MBI: Maslach Burnout Inventory
PD: program director
